# Disentangling the multigenerational transmissions of socioeconomic disadvantages and mental health problems by gender and across lineages: Findings from the Stockholm Birth Cohort Multigenerational Study

**DOI:** 10.1016/j.ssmph.2023.101357

**Published:** 2023-02-07

**Authors:** Baojing Li, Ylva B. Almquist, Can Liu, Lisa Berg

**Affiliations:** Centre for Health Equity Studies (CHESS), Karolinska Institutet/Stockholm University, Department of Public Health Sciences, Stockholm University, SE-106 91, Stockholm, Sweden

**Keywords:** Multigenerational transmission, Socioeconomic conditions, Low income, Mental health, Psychiatric disorders, Longitudinal

## Abstract

There is a paucity of research examining the patterning of socioeconomic disadvantages and mental health problems across multiple generations. The current study therefore aimed to investigate the interconnected transmissions of socioeconomic disadvantages and mental health problems from grandparents to grandchildren through the parents, as well as the extent to which these transmissions differ according to lineage (i.e., through matrilineal/patrilineal descent) and grandchild gender. Drawing on the Stockholm Birth Cohort Multigenerational Study, the sample included 21,416 unique lineages by grandchild gender centered around cohort members born in 1953 (parental generation) as well as their children (grandchild generation) and their parents (grandparental generation). Based on local and national register data, socioeconomic disadvantages were operationalized as low income, and mental health problems as psychiatric disorders. A series of path models based on structural equation modelling were applied to estimate the associations between low income and psychiatric disorders across generations and for each lineage-gender combination. We found a multigenerational transmission of low income through the patriline to grandchildren. Psychiatric disorders were transmitted through both the patriline and matriline, but only to grandsons. The patriline-grandson transmission of psychiatric disorder partially operated via low income of the fathers. Furthermore, grandparents' psychiatric disorders influenced their children's and grandchildren's income. We conclude that there is evidence of transmissions of socioeconomic disadvantages and mental health problems across three generations, although these transmissions differ by lineage and grandchild gender. Our findings further highlight that grandparents' mental health problems could cast a long shadow on their children's and grandchildren's socioeconomic outcomes, and that socioeconomic disadvantages in the intermediate generation may play an important role for the multigenerational transmission of mental health problems.

## Highlights

• Multigenerational transmission of low income and psychiatric disorders is gendered.
• Low income is transmitted through the patriline to grandchildren.
• Psychiatric disorder is transmitted through both lineages but only to grandsons.
• Grandparents' psychiatric disorders predict low income in subsequent generations.
• Low income partly explains multigenerational transmission of psychiatric disorders.

## 1 Introduction

Past research has established the intergenerational transmission (i.e., the association between two generations) of socioeconomic conditions and mental health, respectively (Carvalho, 2012; Eley et al., 2015; Hu & Bobak, 2018; Lundborg et al., 2018). Although research on the multigenerational transmission (i.e. associations between more than two generations) has accelerated in recent years, with a growing body of studies providing evidence for multigenerational associations in either socioeconomic conditions or mental health (Anderson et al., 2018; Daw et al., 2020; Hancock et al., 2013; Mare, 2011, 2014; Pfeffer, 2014), there is a lack of studies investigating these lines of transmission *simultaneously*. Moreover, this strand of research has not sufficiently addressed the issue of gender differences, despite the common notion of life courses being gendered (Hagestad & Dykstra, 2016; Moen, 2001; Rossi, 2018). The significance of research on multigenerational processes is based on a concern with if and how (dis)advantages are generated and sustained across generations, and how socioeconomic, mental health, and gender inequalities evolve over a longer period of time (Lindahl et al., 2015; Mare, 2011; Song et al., 2020). The aim of the current study is therefore to jointly explore these patterns across three generations of Swedish men and women born across the 20th century.

### 1.1 The interplay between socioeconomic conditions and mental health across generations

Socioeconomic disadvantages and mental health problems tend to co-occur within each generation (Hardie & Landale, 2013; Roy & Raver, 2014). It remains uncertain whether evidence is sufficient to assume causality and, if so, in which direction the influences may primarily operate (McLeod & Kaiser, 2004; Schoon et al., 2002). The uncertainty of directionality further convolutes the study of intergenerational associations (Molarius et al., 2009; Santric-Milicevic et al., 2016; Sareen et al., 2011). On the one hand, parental mental health problems can potentially explain the transmission of socioeconomic disadvantages from parents to children, as findings from several longitudinal studies have suggested that parental mental illness is associated with offspring's childhood socioeconomic adversity (Pierce et al., 2020) and adult socioeconomic attainment (Johnston et al., 2013; Slominski et al., 2011). On the other hand, parental socioeconomic disadvantages could play a role in the intergenerational transmission of mental health problems. For instance, children exposed to maternal depression have better mental health outcomes when having a more advantaged socioeconomic situation, including high levels of family income, parental education, and financial support (Goodman, 2020; Pearson et al., 2013).

While the interplay between socioeconomic disadvantages and mental health problems can be difficult to disentangle when only considering two generations, applying a multigenerational perspective adds to the complexity. It is yet unclear whether mental health problems matter for the transmission of socioeconomic disadvantages across multiple generations, and whether socioeconomic disadvantages play a role in the multigenerational transmission of mental health problems.

### 1.2 The role of lineages and gender

Based on previous findings from inter- and multigenerational studies (Andreas et al., 2018; Johnston et al., 2013; Kendler et al., 2018; Pearson et al., 2019), there are reasons to expect the transmission of mental health problems to differ by gender and across lineages. It is nonetheless not apparent how one should characterize these differences. Some research has demonstrated a stronger transmission across the maternal lineage as compared to the transmission from paternal antecedents (Johnston et al., 2013; Pearson et al., 2019) while other studies have concluded that transmission seems to generally be more pronounced for same-sex compared to opposite-sex lineages (Andreas et al., 2018; Kendler et al., 2018). The underlying mechanisms for these findings can primarily be attributed to genes (Anglin et al., 2012; Bergemann & Boles, 2010; Jami et al., 2021), the environment (Natsuaki et al., 2014), or interactions between genetic and environmental factors (Seabrook & Avison, 2010). The findings on differential transmission of socioeconomic disadvantages by gender and across lineages are also largely inconsistent (Neidhöfer & Stockhausen, 2019). However, there seems to be some evidence of a stronger transmission of social class, educational attainment, and earnings on the patrilineal side (Engzell et al., 2020; Hällsten, 2014; Lindahl et al., 2015; Modin et al., 2013). This might be understood in light of the historical dominance of the male breadwinner norm (Gonalons-Pons & Gangl, 2021). Furthermore, the relevance of additional transgenerational epigenetic pathways is supported by recent research showing that effects of ancestral exposure to adverse environment such as food deprivation and low income may be passed down through patriline but not matriline (Pembrey et al., 2006; Vågerö et al., 2018, 2022). In sum, examinations into the interconnected transmissions of socioeconomic disadvantages and mental health problems should consider potential differences by gender and across lineages. Such an assessment may additionally shed some light on the persistence of gender inequalities across generations.

### 1.3 Low income and psychiatric disorders

Applying a multigenerational perspective on the associations between socioeconomic disadvantages and mental health problems is demanding with regard to both study design and data sources. One key challenge is to identify indicators that are sufficiently similar across generations. For this reason, the current study operationalizes socioeconomic disadvantages as low income (at or below the 25th percentile), which is a straightforward indicator of lack of material resources. This indicator has the advantage of being more comparable over time when contrasted with other common indicators of socioeconomic conditions such as educational level or occupational position. For example, graduating from upper secondary school, or being an industrial worker, might mean something different across generations in the context of educational expansion and technological revolutions (Breen, 2010; Maas & van Leeuwen, 2002). Further, to harmonize the measures of mental health problems, we choose to focus on psychiatric disorders. While only a rather unspecific indicator was available for our grandparental generation (encompassing a variety of aspects, such as alcohol abuse, symptoms of depression or psychiatric problems, and receiving psychiatric treatment), it reflects a comparable level of severity of psychiatric disorders as captured in the hospitalization records for the subsequent generations of parents and grandchildren.

### 1.4 The Swedish context

Sweden has experienced vast changes in income inequality over the past 150 years; from being one of the most unequal societies in the industrialized world during the 1870s towards experiencing a great decline in inequality up until the 1970s (Bengtsson & Molinder, 2022). These changes were mostly due to the relative gains among the lower- and middle-income groups driven by the upgrading of the jobs structure, the destruction of capital in the Depression or the Second World War, and the expanding welfare state (Bengtsson & Molinder, 2021, pp. 1909–1950; Gustavsson et al., 2009). Contemporary patterns of socioeconomic and gender differences in mental health in Sweden have nonetheless persisted since the 1950s. Risks of psychiatric disorders became more common in low socioeconomic groups in the 1950s, which marked a shift from the early 1900s (Junkka et al., 2020). This shift is possibly due to that women who constituted the larger share of low socioeconomic groups experienced changing patterns of employment, and consequently a double burden of responsibilities for both work and family, as well as increasing exposure to stress combined with insufficient policy support in the earlier years of the welfare state (Therborn, 2004). In the 1970s, welfare policies on part-time work, publicly financed child care, and parental leave made it easier for mothers to reconcile work and family (Nyberg, 2012), and tax reforms for earned income from a spouses' joint tax system to an individual tax system enhanced the work incentives for wives (Selin, 2014). Consequently, women's labor participation surged in the 1970s. However, in the 1990s, Sweden underwent a severe economic crisis. The process of restructuring the welfare state towards recommodification led to rising unemployment rate, income inequality, and psychological distress, with the greatest increase of levels of nervousness and anxiety among women (Farrants & Bambra, 2018; Persson et al., 2006). Against this background, investigating the associations between low income and psychiatric disorders across generations spanning over a century with a gender perspective may provide us with a more nuanced picture of the complex process of reproduction of inequalities (or mobility) in the Swedish society.

### 1.5 Study aim

The current study aims to disentangle the complexity through which low income and psychiatric disorders may be jointly transmitted across three generations of Swedish men and women born across the 20th century. We address the following research questions:

1. To what extent are low income and psychiatric disorders, respectively, transmitted across three generations (i.e., grandparents, parents, and grandchildren)?
2. In what way, if any, does the multigenerational transmission of psychiatric disorders matter for the multigenerational transmission of low income?
3. In what way, if any, does the multigenerational transmission of low income matter for the multigenerational transmission of psychiatric disorders?
4. Do these above-mentioned transmissions differ by lineage and grandchild gender?

## 2 Materials and methods

### 2.1 Data sources

Data were extracted from the Stockholm Birth Cohort Multigenerational Study (SBC Multigen), which is based on two de-identified data materials: RELINK53 and the Stockholm Metropolitan Study (SMS) (Almquist et al., 2019). RELINK53 comprises all individuals born in 1953 who lived in Sweden in 1960, 1965 and/or 1968, as well as their multigenerational linkages (n = 2,390,753), and contains register-based information from the 1960s up until today. The SMS is defined as all individuals born in 1953 who resided in the greater Stockholm metropolitan area in 1963 (n = 15,117) and contains survey and register-based information from 1953 up until the mid-1980s. By probability matching RELINK53 to the SMS, 14,608 individuals (men: n = 7, 447; women: n = 7, 161; birth year: 1953) could be positively matched and thereby included in the SBC Multigen. These individuals comprise our Generation 1 (G1; parental generation). The SBC Multigen also includes information for the parents of G1, here referred to as Generation 0 (G0; grandparental generation) (n = 29,216; men: n = 14,608; women: n = 14,608; birth years: 1877–1941), and register linkages to the children of G1, referred to as Generation 2 (G2; grandchild generation) (n = 24,929; men: n = 12,848; women: n = 12,081; birth years: 1968–2016). The Regional Ethical Review Board in Stockholm approved the creation of RELINK53 and the SBC Multigen (no. 2017/34–31/5; 2017/684–32). Ethical approval of the current study was granted by the Swedish Ethical Review Authority (no. 2021-00090).

### 2.2 Variables

#### 2.2.1 Low income

For G2, we used information on individual gross income per year from the Longitudinal Integration Database for Health Insurance and Labour Market Studies (part of RELINK53, covering the period 1990–2015). For the majority of G2 (61%), we measured their income at age 30. For those who had not yet turned 30 by 2015 (but did so between 2016 and 2020), we performed imputation using the most recent available income data across ages 25 to 29 (n = 5,778). Based on the same data source, we measured G1's income in 1990 (at age 37 since all G1s were born in 1953). For G0, we used information on earned income in 1963 (when G1 were 10 years old), obtained from the Register of population and income (part of the SMS, covering the period 1953–1985). We subsequently categorized the income data into sex-specific quartiles among all the three generations (also for G2, this categorization was based on the income distribution of those born in the same year). Finally, we created the binary variable “Low income” (Yes/No) by categorizing all individuals into two groups at or below (including individuals who had no income) versus above the lowest 25th percentile.

#### 2.2.2 Psychiatric disorders

G1 and G2 individuals were defined as having psychiatric disorders if they had records of inpatient care with a main or contributing diagnosis reflecting mental and behavioral disorders (Chapter F in the International Classification of Diseases, 10th revision [ICD 10], and the corresponding chapters in the 9th revisions [ICD 9]). Psychiatric disorders with an early onset and typically with a strong biological heritage (ICD 10: F70-F98 and corresponding ICD 9 codes, e.g., intellectual disabilities and developmental disorders) were coded as psychiatric disorders only if there were other co-occurring diagnosis of mental and behavioral disorders. Among G1 and G2 individuals who had psychiatric disorders with an early onset, 58% and 72% also had records of psychiatric inpatient care with other diagnoses. For G1 and G2, information on diagnoses were derived from the National Patient Register (part of RELINK53) for a total period of six years, from three years before income was measured until three years after income was measured (for G1 between age 34 to 40, for G2 the age span varied according to birth year). Corresponding to measures of psychiatric disorders for G1 and G2, information on psychiatric disorders in G0 was derived from two periods (I = 1953–1959 and II = 1960–1965) of the locally kept Social Registers (part of the SMS), which included pre-categorized information about alcohol abuse, psychiatric problems, depression, as well as psychiatric treatment. Among all three generations, psychiatric disorders were coded into binary variables (Yes/No). Details regarding time of measurement for both income and psychiatric disorders for G1 and G2 are given in Figure A1 (a, b).

### 2.3 Analytical sample

**Fig. 1:**
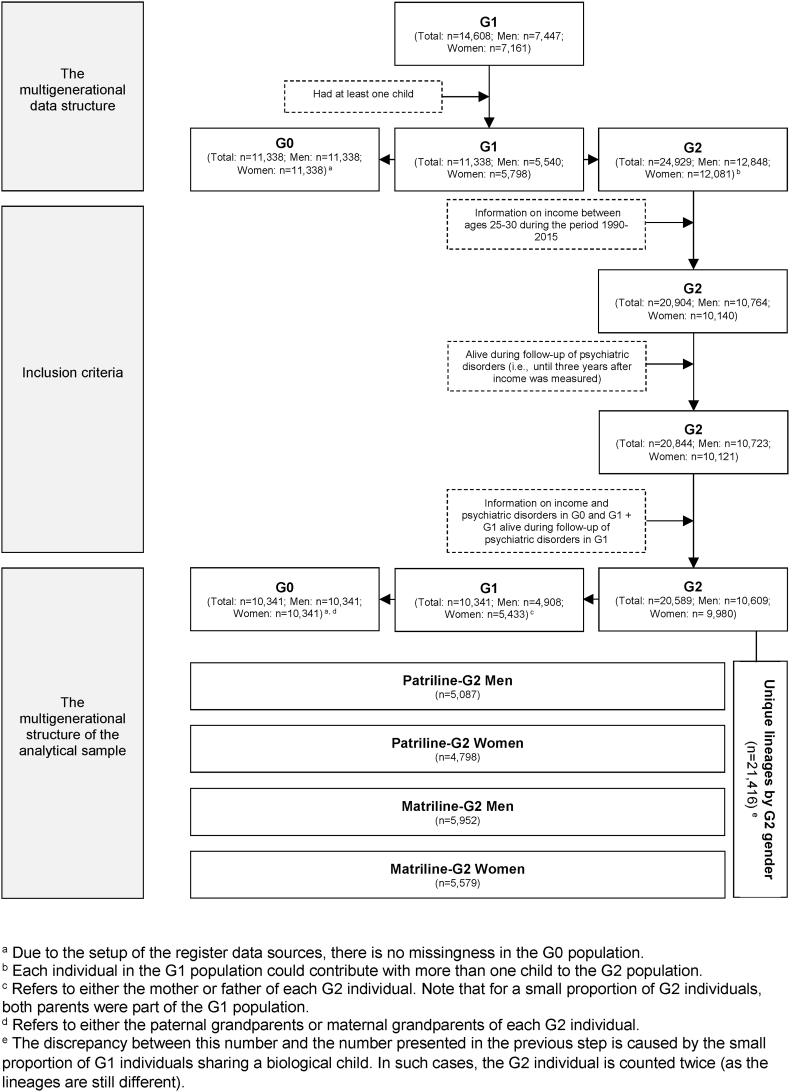
Flowchart of the process by which the analytical sample was selected.

For the current study, the main analytical sample included those of the 24,929 G2 individuals who met a list of inclusion criteria. To be included they had to: 1) have information on income at some point between ages 25 and 30 during the period 1990–2015; 2) be alive during the follow-up of psychiatric disorders (i.e., at least until three years after income was measured); and 3) have information on income and psychiatric disorders in G0 and G1 (G1 additionally had to be alive during the follow-up of psychiatric orders, i.e., at least until three years after their income was measured). The analytical sample consisted of 21,416 unique lineages by G2 gender, including G2 individuals born between 1968 and 1990 (5,087 men and 4,798 women on the patrilineal side and 5,952 men and 5,579 women on the matrilineal side), and their parents and grandparents from G1 and G0. The distribution of birth years in the analytical sample was as follows: 1877–1941 for G0, 1953 for G1, and 1968–1990 for G2. More details of the process by which the analytical sample was derived are shown in Fig. 1.

### 2.4 Statistical analysis

Structural equation modelling (SEM) was used to estimate the associations between low income and psychiatric disorders across the three generations. SEM allows for continuous, binary, categorical, and ordered measures to be modelled using linear, logistic, multinomial, and ordinal logistic specifications, respectively.

One common approach in path analysis is to set up a series of alternative models. Model 1 includes only so-called autoregressive paths (in the current study, they reflect the multigenerational transmission of low income and psychiatric disorders, respectively, from G0 to G1 and from G1 to G2). Model 2 additionally includes cross-lagged paths going from low income to psychiatric disorders across generations, whereas Model 3 instead reflects the opposite direction, with cross-lagged paths going from psychiatric disorders to low income across generations. Model 4 encompasses all paths specified in the previous models. We used this step-wise approach to study what happens when additional paths are added.

Each model was performed with the four lineage-G2 gender combinations (a. patriline-G2 men: paternal grandfather-father-son, b. patriline-G2 women: paternal grandfather-father-daughter, c. matriline-G2 men: maternal grandmother-mother-son, and d. matriline-G2 women: maternal grandmother-mother-daughter) as grouping variables. In Model 4, we tested whether the path estimates were statistically significantly different (p < 0.05) across the four lineage-G2 gender combinations, using Wald tests. As for sensitivity analyses, we re-ran the models 1) for the sample with alternative coding strategy for G0 women, and 2) for the sample without income imputation for G2.

All our models additionally included the correlation between low income and psychiatric disorders in G0 that can be jointly influenced by common unobserved background factors (e.g., genetic factors or unobserved characteristics of the family). The parameters were estimated using the maximum likelihood with robust standard errors, which is robust for non-normally distributed data (binary variables) (Finney & DiStefano, 2006, pp. 269–314), and estimates were presented with exponentiated coefficients (i.e. Odds Ratios [OR]). Data were managed using Stata SE 16, and all analyses were performed in Mplus Version 8.

## 3 Results

Table 1 summarizes the information on low income and psychiatric disorders for G2 men and women as well as for their parents (G1) and grandparents (G0), stratified by lineage. As expected, approximately 25% of G0, G1 and G2 had low income. The exception was G0 women, where around 50% were in the low-income group (this is because this category additionally includes those registered as no income; this was relatively common among G0 women). Approximately 2–4% of G0, G1, and G2 individuals had experience of inpatient care due to psychiatric disorders. Substance use-related disorders, followed by mood disorders and anxiety disorders, were the most common diagnoses in all groups except for G2 women on both patrilineal and matrilineal side for whom mood disorders were more common than substance-related disorders (Table A1 and Table A2).

**Table 1:** Distribution of low income and psychiatric disorders in G2 (grandchildren), G1 (parents of G2), and G0 (grandparents of G2), stratified by lineage and G2 gender.

	Patriline						Matriline					
	G2 Men (grandsons)			G2 Women (granddaughters)			G2 Men (grandsons)			G2 Women (granddaughters)		
	Grandfathers n (%)	Fathers n (%)	Grandsons n (%)	Grandfathers n (%)	Fathers n (%)	Granddaughters n (%)	Grandmothers n (%)	Mothers n (%)	Grandsons n (%)	Grandmothers n (%)	Mothers n (%)	Granddaughters n (%)
Total	5,087	5,087	5,087	4,798	4,798	4,798	5,952	5,952	5,952	5,579	5,579	5,579
**Low income**												
No	3,797 (74.6)	3,948 (77.6)	3,823 (75.2)	3,636 (75.8)	3,812 (79.5)	3,640 (75.9)	3,014 (50.6)	4,281 (71.9)	4,457 (74.9)	2,780 (49.8)	4,011 (71.9)	4,165 (74.7)
Yes	1,290 (25.4)	1,139 (22.4)	1,264 (24.9)	1,162 (24.2)	986 (20.6)	1,158 (24.1)	2,938 (49.4)	1,671 (28.1)	1,495 (25.1)	2,799 (50.2)	1,568 (28.1)	1,414 (25.4)
**Psychiatric disorders**												
No	4,860 (95.5)	4,939 (97.1)	4,908 (96.5)	4,591 (95.7)	4,684 (97.6)	4,624 (96.4)	5,787 (97.2)	5,797 (97.4)	5,755 (96.7)	5,428 (97.3)	5,425 (97.2)	5,371 (96.3)
Yes	227 (4.5)	148 (2.9)	179 (3.5)	207 (4.3)	114 (2.4)	174 (3.6)	165 (2.8)	155 (2.6)	197 (3.3)	151 (2.7)	154 (2.8)	208 (3.7)

**Fig. 2:**
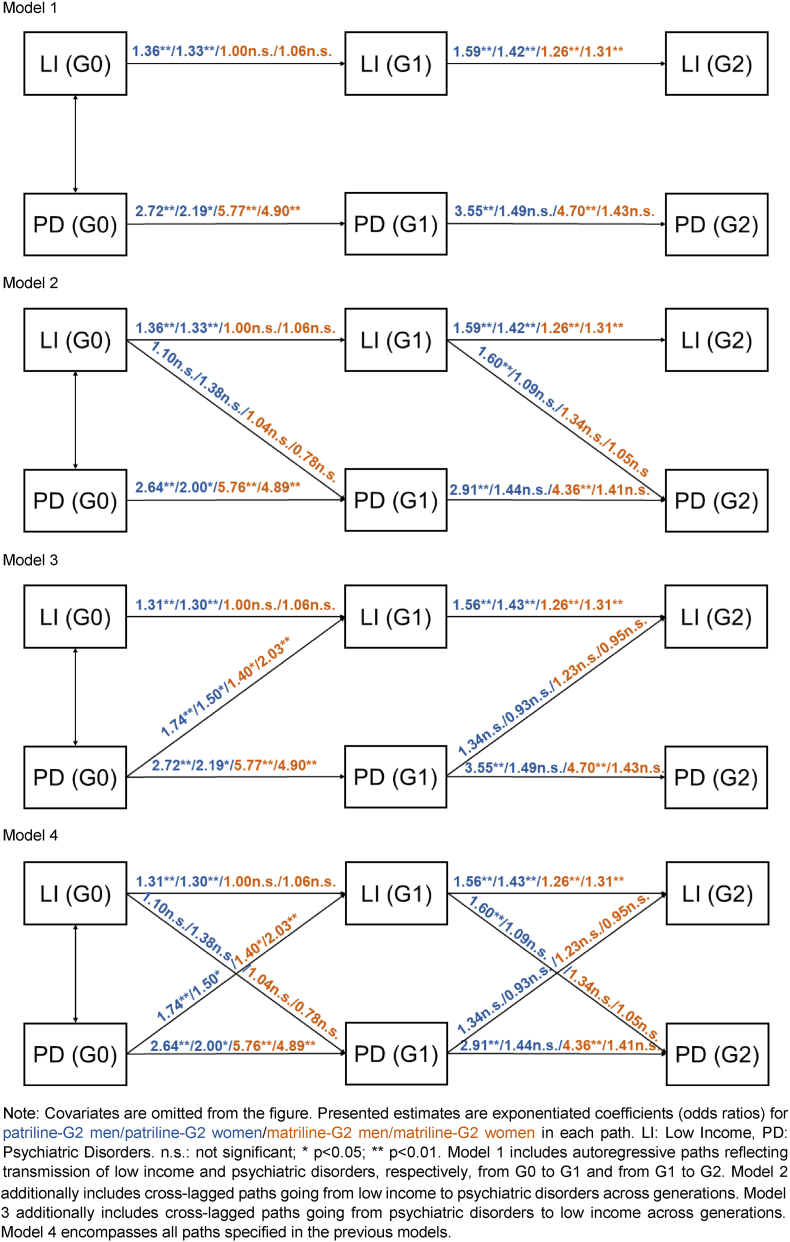
Structural equation models for the multigenerational transmissions of low income and psychiatric disorders, by lineage and grandchild gender.

Fig. 2 presents ORs from Models 1–4 (for ORs with 95% confidence interval, see Table A3). Model 1 shows that, on the patrilineal side, low income transmitted across the three generations irrespective of G2 gender. As for the matrilineal side, low income only transmitted from G1 to G2 irrespective of G2 gender. In terms of psychiatric disorders, the transmission through both patriline and matriline seemed to be more evident among G2 men than G2 women and was tested to be stronger on the matrilineal side (Table A4).

Model 2 shows that the paths from low income to psychiatric disorders in subsequent generations were generally not statistically significant. An exception was low income in G1 to psychiatric disorders in G2 men through patriline, which contributed to the path from low income in G0 to low income in G1 and further on to psychiatric disorders in G2 men.

Model 3 shows paths going from psychiatric disorders in G0 to low income in G1 were statistically significant irrespective of lineage and G2 gender, contributing to the paths from psychiatric disorders in G0 to low income in G1 and further on to low income in G2. However, the corresponding paths going from psychiatric disorders in G1 to low income in G2 were not statistically significant.

Model 4 integrated all previous models and additionally shows that low income in G1 partly explained the link between psychiatric disorders in G0 and psychiatric disorders in G2 men through patriline. By contrast, psychiatric disorders in G1 did not seem to explain the link between low income in G0 and low income in G2.

In the sensitivity analyses without income imputation, results were similar to those from the main analyses, with a few exceptions: 1) for patriline-G2 women, the path from psychiatric disorders in G0 to low income in G1 was no longer statistically significant; and 2) for matriline-G2 men, the path from psychiatric disorders in G1 to low income in G2, and the path from low income in G1 to psychiatric disorders in G2 turned statistically significant (Figure A2).

## 4 Discussions

This study sought to disentangle the multigenerational transmissions of low income and psychiatric disorders. Below, the key findings are discussed under separate headings.

### 4.1 Low income is transmitted across the patriline

Our results showed that there was a multigenerational transmission of low income across the patriline; from grandfathers to fathers to grandchildren. This is consistent with previous intergenerational studies on fathers' and children's earnings (Becker & Tomes, 1986; Fertig, 2001; Haider & Solon, 2006; Solon, 1999). Given that our operationalization of income was individual gross income obtained through employment, this result might be interpreted based on occupational theories such as ‘occupational following’ and the ‘role model hypothesis’ (Hill & Duncan, 1987; Laband & Lentz, 1983). These theories posit that sons grow up and follow their fathers' occupational footsteps in, for example, business, politics, the arts, and agriculture. The father's labor income reflects the quality of the labor market role model and can be expected to have a positive effect on the child's labor market qualifications and attainments, especially if the child is a son (Hill & Duncan, 1987; Laband & Lentz, 1983). While most empirical studies have focused predominately on the stability of labor market outcomes between fathers and sons, it has been unclear whether fathers and daughters would have the similar pattern of occupational following. In the current study, our main analyses showed no multigenerational transmission for the matriline. During the 1950s – a period characterized as the Swedish ‘housewife era’ – the paid worked hours of women reached a low point (Edvinsson & Edvinsson, 2017). This was followed by a rapid growth in female labor force participation rates in Sweden during the post-war era, especially among married women (Selin, 2014). This might, to some extent, explain why we did not observe a significant transmission of individual-level low income from grandmothers to the intermediate generation of mothers. However, these results do not necessarily mean that there is no association between low income in grandmothers and low income in mothers when household-level income is considered. To further investigate this issue, we performed a sensitivity analysis measuring household-level income among grandmothers who were housewives and whose husband was in the low income (No) group, and they were recategorized from low income (Yes) to low income (No) group. The results from this sensitivity analysis suggest that there is a transmission from household-level low income in grandmothers to low income in mothers (see Figure A3). Given the mixed results on the transmission of low income from grandmothers to mothers by individual-versus household-level grandmother income, evidence is still unclear regarding whether the transmission of low income operated across the matriline. Future research may pursue a better understanding on this issue through investigating the role of marital status or alternative socioeconomic indicators in such multigenerational transmission.

### 4.2 There is a multigenerational transmission of psychiatric disorders among grandsons

A multigenerational transmission of psychiatric disorders was seen only for grandsons. One potential explanation could be that our operationalization of psychiatric disorders captures primarily substance-related disorders, and men are more likely to be affected by these disorders than women (Rolando et al., 2020). However, further investigations that include many other factors (e.g., intra- and extra-familial influences such as parental separation, and relationships with peers (Evans et al., 2013; Jobe-Shields et al., 2015)) are needed to provide a better understanding on the mechanisms underlying this gender difference in the multigenerational transmission of psychiatric disorders. Interestingly, for grandsons, a stronger effect was found in the transmission of psychiatric disorders on the matrilineal side. This also corroborates previous findings; for example, a previous study found that when mothers were hospitalized in psychiatric care, children suffered from mood and emotional disorders, behavioral disorders, and schizophrenia more than when fathers were hospitalized (Paananen et al., 2021). Similarly, results from another study indicated that offspring substance use is significantly influenced by both mothers and grandmothers, but not by fathers or grandfathers (Thornberry et al., 2006). This could be explained by several influential theories of child development, including psychodynamic theories and attachment theories, which emphasize the key role of mothers in their children's early development and socialization (Bowlby, 1997). Moreover, it must be pointed out that while we include register data for both biological parents, it is not possible to discern whether the child was actually living with both of them and for how long. The transmission expected to operate via shared environment might accordingly be underestimated, particularly for grandfathers and fathers since it is more common that children stay with their mother in cases of parental separation or divorce (Mok et al., 2018). Future studies with the possibility to investigate differences in the transmission when it comes to different family types, such as nuclear families, single-parent families, and extended families, would provide a better understanding of the mechanisms underlying the multigenerational transmission of psychiatric disorders.

### 4.3 Grandparents' psychiatric disorders are associated with their children's and grandchildren's income

The results showed that psychiatric disorders among grandparents mattered for the transmission of low income from parents to grandchildren. This is in line with previous intergenerational research which has demonstrated that parental mental health problems contribute to offspring's socioeconomic outcomes (Pierce et al., 2020; Slominski et al., 2011), even in the context of the extensive Swedish welfare system (Pierce et al., 2020). A unique contribution of our study is that we expanded into a multigenerational approach by investigating the role of grandparental mental health problems in the transmission of socioeconomic disadvantages across generations. One possible explanation of our findings is that children of parents with mental health problems are more likely to experience multiple challenges throughout the life course compared with other children, such as separation from parents (Ranning et al., 2016), and less than optimal parenting (Paulson et al., 2006). These challenges put children at higher risk of negative social outcomes, such as school dropout (Shen et al., 2016) and teenage pregnancy (Anda et al., 2002), which would in the long run influence their own and even their children's socioeconomic outcomes across the life course.

### 4.4 Low income plays a role for the multigenerational transmission of psychiatric disorders among grandsons, whereas psychiatric disorders do not seem to matter for the multigenerational transmission of low income

Another key finding of this study is that the multigenerational transmission of psychiatric disorders from grandfathers to grandsons partially operated via low income in the intermediate generation of fathers. Similarly, previous research has found that mental health problems are more prevalent in disadvantaged families, and the persistence of mental health problems across generations is likely to be linked to these inherited family contexts due to individuals' experience of material deprivation at an early age (Lorant et al., 2003; McLaughlin et al., 2011; Reiss, 2013). Our results particularly highlight the importance of fathers in the transmission of psychiatric disorders across generations. Even with the more egalitarian gender ideology of today, work identity and the traditional role of primary earner may still be critical issues for fathers (Kramer & Pak, 2018). As can be seen in previous research, a decrease in one's share of family income over time is related to an increase in the level of depressive symptoms among fathers and a decrease in the level of depressive symptoms among mothers (Kramer & Pak, 2018). Accordingly, low income among fathers might be more likely to exacerbate their mental health problems compared to low income among mothers, and might further have negative consequences for their children's mental health. By contrast, psychiatric disorders among parents was not a mediating pathway for the multigenerational transmission of low income from grandparents to grandchildren. The income of grandchildren was measured at a relatively earlier life stage between 25 and 30, and research has shown that those with high lifetime income tend to have low income at the beginning of their career (Nybom & Stuhler, 2017). Thus, the measurement of grandchildren's income may lead to its association with the intermediate generation of parents' psychiatric disorders to be understated. Particularly for the matrilineal side, the low work force participation rates during the 1950s (Edvinsson & Edvinsson, 2017) might, to some extent, explain why on the matrilineal side low income among grandmothers was not significantly associated with psychiatric disorders in the intermediate generation of mothers, and consequently, not showing the same patterns of mediation.

### 4.5 Strengths and limitations

A major strength of this study is that it builds on a multigenerational material that encompasses local and national register data for a relatively large, community-based sample. This made it possible to measure low income and psychiatric disorders in three subsequent generations in a largely comparable way, as well as to examine differences across lineages and by grandchild gender.

Some important limitations should nonetheless be considered. First of all, despite that the data material has a multigenerational structure, we only have G0 linkages for the G1 individuals who were part of the original SMS cohort born in 1953. Second, with regard to income, it was measured between ages 25 and 30 for G2. This likely serves as a suboptimal indication of lifetime income as research has shown that those with high lifetime income tend to have low income at the beginning of their career (Nybom & Stuhler, 2017). Accordingly, we risk underestimating the strength of the correlation between G1 income and G2 income. While a previous Swedish study using the same data material showed that G1 income quintiles were largely stable between ages 25–29 compared to age 37 (Jackisch & van Raalte, 2022), it is still uncertain whether this finding could also be extended to the G2 in our study. However, we conducted sensitivity analyses where we included only those G2 individuals for whom we had income information at age 30, and only minor differences compared to the results from the main analysis were noted. Another issue that needs to be discussed is the choice to use the G0 women's own income, a choice that reflects the key ambition – and one of the unique contributions – of the current study, that is to retain a clear division between patrilineal and matrilineal sides. However, since there was a large proportion of housewives among G0 women, we performed a sensitivity analysis to capture the economic welfare on the household level among G0 women to complement our main analyses of the individual-level transmission of low income across generations (see Figure A3).

Other limitations concern our measures of psychiatric disorders. Since many psychiatric disorders are considered to have a hereditary component (Abkevich et al., 2003; Dick et al., 2003; Singh et al., 2011), one general limitation is that we have no access to genetic information which makes it impossible to assess to what extent genes contribute to the multigenerational associations. Moreover, for G1 and G2, information on psychiatric disorders was based on hospitalization data from the National Patient Register. Thus, we are only able to capture the most severe cases, which may lead to underestimation of the prevalence of these conditions in the population. Due to the small proportion of individuals being hospitalized as having psychiatric disorders, we were not able to examine cause-specific outcomes which may potentially provide deeper insights into possible mechanisms.

Another relevant issue concerns the generalizability of the results when it comes to later cohorts: having psychiatric disorders may have meant something different for G0, G1, and G2. Worth noting is that a major “psychiatry reform” was introduced in Sweden in 1995, seeking to improve the living situations for the mentally ill people with efforts from both the social services and psychiatric care, thereby promoting social integration and reducing stigma (Arvidsson, 2004). Despite this reform, however, and the fact there have been changes of diagnostic criteria (i.e., in 1987 when ICD-9 was introduced, and in 1997 when ICD-10 was introduced), the number of hospital discharges in the inpatient register has remained stable since 1973 (Ludvigsson et al., 2011). With regard to G0, the indicator of psychiatric disorders was derived based on registrations of alcohol abuse, depression or psychiatric problems, and psychiatric treatment in the Social Registers (since the National Patient Register only commenced in 1964 and did not have sufficient and nationwide coverage until 1973). Such inconsistency in measurement may explain some of the variations in the strength of the correlations between the different generations.

### 4.6 Future research

Even though low income and psychiatric disorders were found to be interconnected across generations, there may be many other factors influencing these interconnected associations. Therefore, future explanatory research is needed to take a wider range of factors into consideration, for example, to investigate how other socioeconomic and health indicators (e.g., education and occupation, early child development, maternal health, and life course determinants) may help break the multigenerational transmission of psychiatric disorders, and how intra- and extra-familial influences (e.g., family structure, marital status, and relationships with peers) may provide insights into potential mechanisms underlying the multigenerational transmissions of low income and psychiatric disorders. It should be emphasized that the primary focus of the current study was not to draw any causal inferences, but rather to investigate whether the associations of low income could be explained by psychiatric disorders and, vice versa, whether the associations of psychiatric disorders could be explained by low income across generations. It is nonetheless an important task for future studies to further inquiry into a causal framework.

## 5 Conclusion

Our findings demonstrated transmissions of socioeconomic disadvantages and mental health problems across three generations, with important differences by matrilineal/patrilineal descent and grandchild gender. The findings further showed that grandparents' mental health problems could cast a long shadow on their children's and grandchildren's socioeconomic outcomes, and that socioeconomic disadvantages of the intermediate generation plays a role for the multigenerational transmission of mental health problems.

## Ethical statement

The Regional Ethical Review Board in Stockholm approved the creation of RELINK53 and the SBC Multigen (no. 2017/34–31/5; 2017/684–32). Ethical approval of the current study was granted by the Swedish Ethical Review Authority (no. 2021-00090).

## CRediT authorship contribution statement

**Baojing Li:** Conceptualization, Methodology, Software, Formal analysis, Writing – original draft, Visualization, and, Project administration. **Ylva B. Almquist:** Conceptualization, Methodology, Writing – review & editing, Supervision, Project administration, and, Funding acquisition. **Can Liu:** Methodology, Writing – review & editing, and, Supervision. **Lisa Berg:** Conceptualization, Methodology, Writing – review & editing, Supervision, and, Project administration.

## Declaration of competing interest

None.

## Data Availability

The authors do not have permission to share data.
