# Supplementary material for: Disentangling the multigenerational transmissions of socioeconomic disadvantages and mental health problems by gender and across lineages: Findings from the Stockholm Birth Cohort Multigenerational Study
Source: SSM Popul Health. 2023 Feb 7;22:101357. doi: 10.1016/j.ssmph.2023.101357 (PMC9947103; doi:10.1016/j.ssmph.2023.101357)
Supplement: Multimedia component 1 [file mmc1.docx]

**Table A1. Occurrence of psychiatric disorders in G2 (grandchildren) and G1 (parents of G2), stratified by lineage and G2 gender.**

|  | **Patriline** | | | | **Matriline** | | | |
| --- | --- | --- | --- | --- | --- | --- | --- | --- |
|  | **G2 Men**  **(grandsons)** | | **G2 Women**  **(granddaughters)** | | **G2 Men**  **(grandsons)** | | **G2 Women**  **(granddaughters)** | |
|  | Fathers  n (%) | Grand-  sons  n (%) | Fathers  n (%) | Grand-  daughters  n (%) | Mothers  n (%) | Grand-  sons  n (%) | Mothers  n (%) | Grand-  daughters  n (%) |
| Total sample, n | 5,087 | 5,087 | 4,798 | 4,798 | 5,952 | 5,952 | 5,579 | 5,579 |
| Organic disorders | / | / | / | / | 3  (0.05) | 1  (0.02) | 2  (0.04) | 3  (0.05) |
| Substance-related disorders | 105  (2.06) | 96  (1.89) | 72  (1.50) | 55  (1.15) | 68  (1.14) | 120  (2.02) | 72  (1.29) | 53  (0.95) |
| Schizophrenic/ Psychotic disorders | 7  (0.14) | 23  (0.45) | 10  (0.21) | 14  (0.29) | 23  (0.39) | 28  (0.47) | 17  (0.30) | 13  (0.23) |
| Mood disorders | 12  (0.24) | 61  (1.20) | 11  (0.23) | 84  (1.75) | 28  (0.47) | 58  (0.97) | 24  (0.43) | 99  (1.77) |
| Anxiety disorders | 13  (0.26) | 36  (0.71) | 9  (0.19) | 49  (1.02) | 9  (0.15) | 34  (0.57) | 9  (0.16) | 72  (1.29) |
| Personality disorders | 5  (0.10) | 11  (0.22) | 17  (0.35) | 21  (0.44) | 24  (0.40) | 16  (0.27) | 29  (0.52) | 25  (0.45) |
| Mental retardation and diseases originating in childhood* | 11  (0.22) | 37  (0.73) | 11  (0.23) | 33  (0.69) | 21  (0.35) | 37  (0.62) | 11  (0.20) | 33  (0.59) |
| Other disorders | 28  (0.55) | 18  (0.35) | 24  (0.50) | 25  (0.52) | 47  (0.79) | 22  (0.37) | 47  (0.84) | 29  (0.52) |

* Cases only included if there are other co-occurring diagnoses of psychiatric disorders.

**Table A2. Psychiatric disorders: ICD-9 and ICD-10 codes.**

| **Disorders** | **Conditions** | **ICD-9 code** | **ICD-10 code** |
| --- | --- | --- | --- |
| Organic disorders | Senile and pre-senile psychotic conditions | 290.0–290.9 | F00–F09, G30 |
|  | Transient organic psychotic conditions | 293.0, 293.1, 293.8, 293.9 |  |
|  | Other organic psychotic conditions | 294.0, 294.1, 294.8, 294.9 |  |
| Substance-related disorders | Alcoholic psychoses | 291.0–291.9 | F10–F19, F55 |
|  | Drug psychoses | 292.0–292.9 |  |
|  | Alcohol dependence | 303.0–303.9 |  |
|  | Drug dependence | 304.0–304.9 |  |
|  | Nondependent abuse of drugs | 305.0–305.9 |  |
| Schizophrenic/ Psychotic disorders | Schizophrenia | 295.0–295.9 | F20–F29 |
|  | Psychotic | 298.8–298.9 |  |
|  | Paranoia | 297.1–297.3 |  |
| Mood disorders | Bipolar | 296.0–296.1, 296.4–296.8 | F30, F31, F34.0 |
|  | Depression | 296.2, 296.3, 300.4, 311 | F32, F33, F34.1, F38.1 |
|  | Other | 296.9 | F34.8, F34.9, 38.0, F38.8, F39 |
| Anxiety disorders | Anxiety | 300.0, 300.2, 300.3, 309.8 | F40, F41, F42, F93.0–F93.2 |
|  | Acute stress | 308.3 | F43.0, 43.1, 43.8, 43.9 |
| Personality disorders | Personality disorders | 301.0–301.9 | F60, F61, F62, F69 |
| Other disorders | Adjustment disorders | 309.0–309.4, 309.8–309.9 | F43.2, F99 |
|  | Sexual disorders | 302.0–302.9 | F52, F64, F65, F66 |
|  | Delusional disorders | 297.0–297.3, 297.8–297.9 |  |
|  | Disturbance of conduct not otherwise specified | 312.0–312.4, 312.8–312.9 | F63, F91, F92, F95 |
|  | Non-organic psychoses | 298.0–298.4 |  |
|  | All other psychiatric disorders | 299.0, 299.1, 299.8, 299.9, 300.1, 300.5, 300.6, 300.7, 300.8, 300.9, 307.0, 307.1, 307.2, 307.3-307.7, 307.9, 308.0-308.2, 308.9, 310, 313.0-313.3, 313.8, 313.9, 314.0-314.2, 314.8, 314.9, 315.0-315.5, 315.9, 316, 317, 318.0-318.2, 319 | F44, F48, F50, F51, F53, F54, F68, F70–F73, F84, F98, F79, F78, F80–F82, F83, F88, F89, F90, F93.3–F93.9, F94 |

Source: Canadian Institute for Health Information, Hospital Mental Health Services in Canada, 2005-2006 Ottawa 2007, p. 27-28 (https://www150.statcan.gc.ca/n1/pub/82-622-x/2011006/tbl/tbla-eng.htm).

**Table A3. Structural equation models for the multigenerational transmissions of low income and psychiatric disorders, stratified by lineage and G2 gender.**

|  | **Paths** | **Patriline-G2 men** | **Patriline-G2 women** | **Matriline-G2 men** | **Matriline-G2 women** |
| --- | --- | --- | --- | --- | --- |
| **Model 1** | LI(G0) → LI(G1) | 1.36 [1.17, 1.57] | 1.33 [1.14, 1.56] | 1.00 [0.90, 1.12] | 1.06 [0.95, 1.20] |
|  | LI(G1) → LI(G2) | 1.59 [1.38, 1.84] | 1.42 [1.22, 1.66] | 1.26 [1.11, 1.44] | 1.31 [1.15, 1.49] |
|  | PD(G0) → PD(G1) | 2.72 [1.59, 4.65] | 2.19 [1.13, 4.26] | 5.77 [3.51, 9.50] | 4.90 [2.88, 8.34] |
|  | PD(G1) → PD(G2) | 3.55 [2.07, 6.11] | 1.49 [0.65, 3.45] | 4.70 [2.87, 7.70] | 1.43 [0.69, 2.96] |
| **Model 2** | LI(G0) → LI(G1) | 1.36 [1.17, 1.57] | 1.33 [1.14, 1.56] | 1.00 [0.90, 1.12] | 1.06 [0.95, 1.20] |
|  | LI(G1) → LI(G2) | 1.59 [1.38, 1.84] | 1.42 [1.22, 1.66] | 1.26 [1.11, 1.44] | 1.31 [1.15, 1.49] |
|  | PD(G0) → PD(G1) | 2.64 [1.53, 4.56] | 2.00 [1.02, 3.92] | 5.76 [3.50, 9.48] | 4.89 [2.87, 8.33] |
|  | PD(G1) → PD(G2) | 2.91 [1.66, 5.09] | 1.44 [0.62, 3.37] | 4.36 [2.65, 7.18] | 1.41 [0.68, 2.93] |
|  | LI(G0) → PD(G1) | 1.10 [0.76, 1.60] | 1.38 [0.92, 2.07] | 1.04 [0.75, 1.43] | 0.78 [0.57, 1.08] |
|  | LI(G1) → PD(G2) | 1.60 [1.15, 2.22] | 1.09 [0.76, 1.58] | 1.34 [0.99, 1.81] | 1.05 [0.77, 1.42] |
| **Model 3** | LI(G0) → LI(G1) | 1.31 [1.13, 1.51] | 1.30 [1.11, 1.52] | 1.00 [0.89, 1.12] | 1.06 [0.95, 1.20] |
|  | LI(G1) → LI(G2) | 1.56 [1.34, 1.81] | 1.43 [1.22, 1.67] | 1.26 [1.10, 1.43] | 1.31 [1.15, 1.49] |
|  | PD(G0) → PD(G1) | 2.72 [1.59, 4.65] | 2.19 [1.13, 4.26] | 5.77 [3.51, 9.50] | 4.90 [2.88, 8.34] |
|  | PD(G1) → PD(G2) | 3.55 [2.07, 6.11] | 1.49 [0.65, 3.45] | 4.70 [2.87, 7.70] | 1.43 [0.69, 2.96] |
|  | PD(G0) → LI(G1) | 1.74 [1.30, 2.31] | 1.50 [1.10, 2.05] | 1.40 [1.01, 1.94] | 2.03 [1.46, 2.82] |
|  | PD(G1) → LI(G2) | 1.34 [0.94, 1.91] | 0.93 [0.60, 1.43] | 1.23 [0.87, 1.75] | 0.95 [0.66, 1.37] |
| **Model 4** | LI(G0) → LI(G1) | 1.31 [1.13, 1.51] | 1.30 [1.11, 1.52] | 1.00 [0.89, 1.12] | 1.06 [0.95, 1.20] |
|  | LI(G1) → LI(G2) | 1.56 [1.34, 1.81] | 1.43 [1.22, 1.67] | 1.26 [1.10, 1.43] | 1.31 [1.15, 1.49] |
|  | PD(G0) → PD(G1) | 2.64 [1.53, 4.56] | 2.00 [1.02, 3.92] | 5.76 [3.50, 9.48] | 4.89 [2.87, 8.33] |
|  | PD(G1) → PD(G2) | 2.91 [1.66, 5.09] | 1.44 [0.62, 3.37] | 4.36 [2.65, 7.18] | 1.41 [0.68, 2.93] |
|  | LI(G0) → PD(G1) | 1.10 [0.76, 1.60] | 1.38 [0.92, 2.07] | 1.04 [0.75, 1.43] | 0.78 [0.57, 1.08] |
|  | LI(G1) → PD(G2) | 1.60 [1.15, 2.22] | 1.09 [0.76, 1.58] | 1.34 [0.99, 1.81] | 1.05 [0.77, 1.42] |
|  | PD(G0) → LI(G1) | 1.74 [1.30, 2.31] | 1.50 [1.10, 2.05] | 1.40 [1.01, 1.94] | 2.03 [1.46, 2.82] |
|  | PD(G1) → LI(G2) | 1.34 [0.94, 1.91] | 0.93 [0.60, 1.43] | 1.23 [0.87, 1.75] | 0.95 [0.66, 1.37] |

Note: Covariates are omitted from the table. Presented estimates are exponentiated coefficients (odds ratios) with 95% confidence intervals. LI: Low Income, PD: Psychiatric Disorders.

**Table A4. Statistical tests of differences between different lineage-G2 gender combinations for each path.**

| **Paths** | **Patriline-G2 men** | **Patriline-G2 women** | **Matriline-G2 men** | **Matriline-G2 women** | **P value (Wald test)** |
| --- | --- | --- | --- | --- | --- |
| LI(G0) → LI(G1) | 0.27 | 0.26 | 0.00 | 0.06 | 0.01 |
| LI(G1) → LI(G2) | 0.44 | 0.36 | 0.23 | 0.27 | 0.14 |
| PD(G0) → PD(G1) | 0.97 | 0.69 | 1.75 | 1.59 | 0.03 |
| PD(G1) → PD(G2) | 1.07 | 0.36 | 1.47 | 0.34 | 0.03 |
| LI(G0) → PD(G1) | 0.10 | 0.32 | 0.04 | -0.25 | 0.18 |
| LI(G1) → PD(G2) | 0.47 | 0.09 | 0.29 | 0.05 | 0.25 |
| PD(G0) → LI(G1) | 0.55 | 0.41 | 0.34 | 0.71 | 0.39 |
| PD(G1) → LI(G2) | 0.29 | -0.07 | 0.21 | -0.05 | 0.43 |

Note: Presented estimates are unexponentiated coefficients from Model 4.

**Figure A1a. Time of measurement for both low income and psychiatric disorders for G2.**


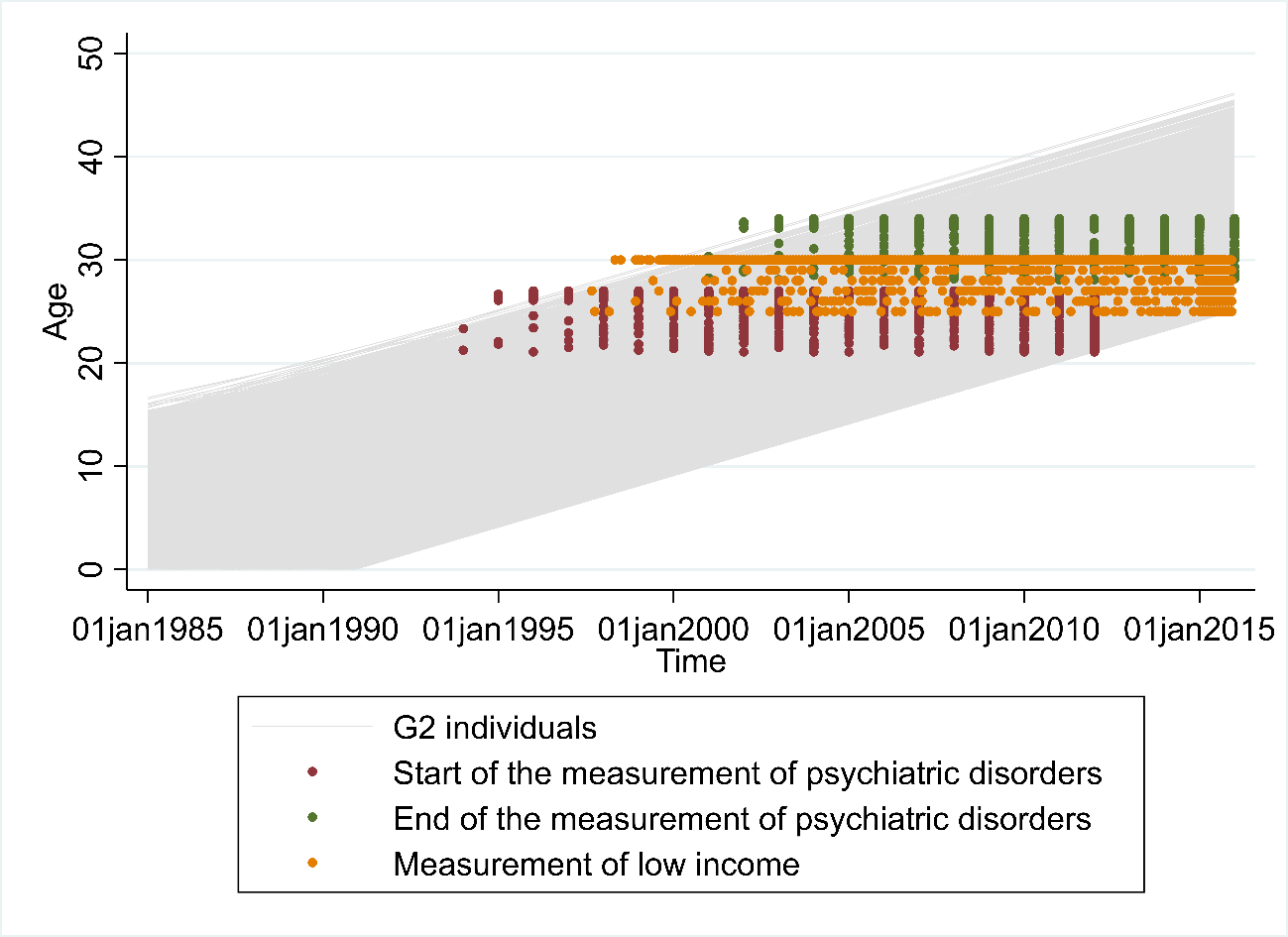


Note: Lexis diagram with dots representing measurement of income and psychiatric disorders at different time points in life, and crosses showing other events occurring across the life course among G2 individuals.

**Figure A1b. Time of measurement for both low income and psychiatric disorders for G1.**


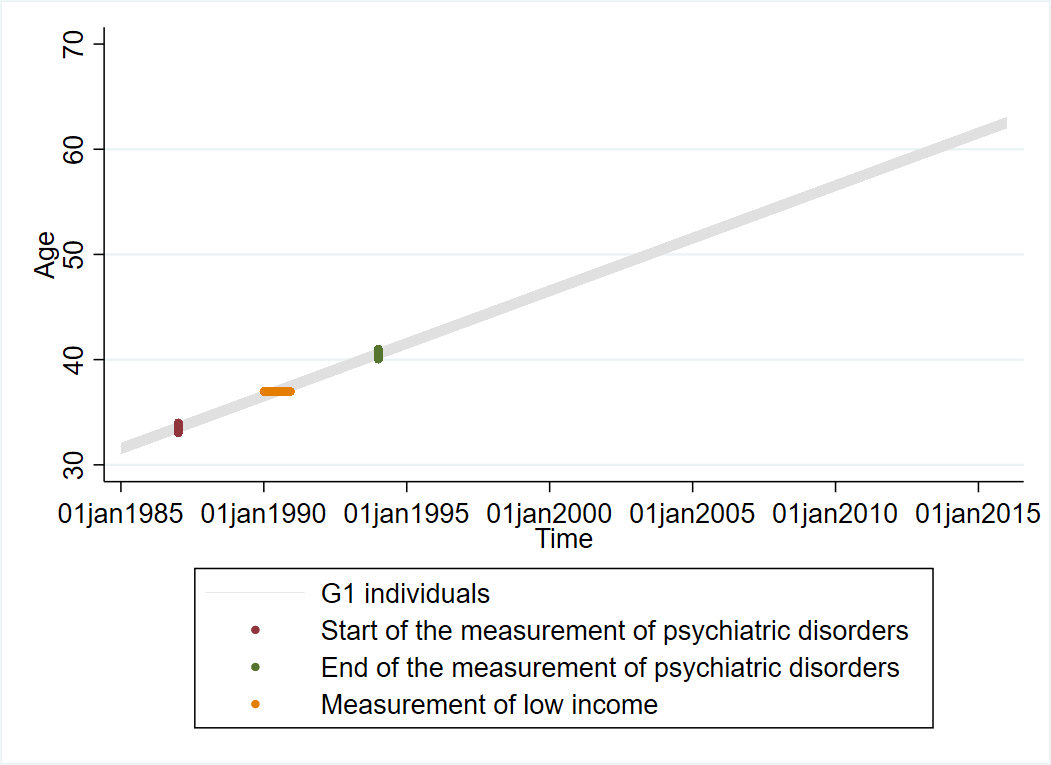


Note: Lexis diagram with dots representing measurement of income and psychiatric disorders at different time points in life, and crosses showing other events occurring across the life course among G1 individuals.

Model 1


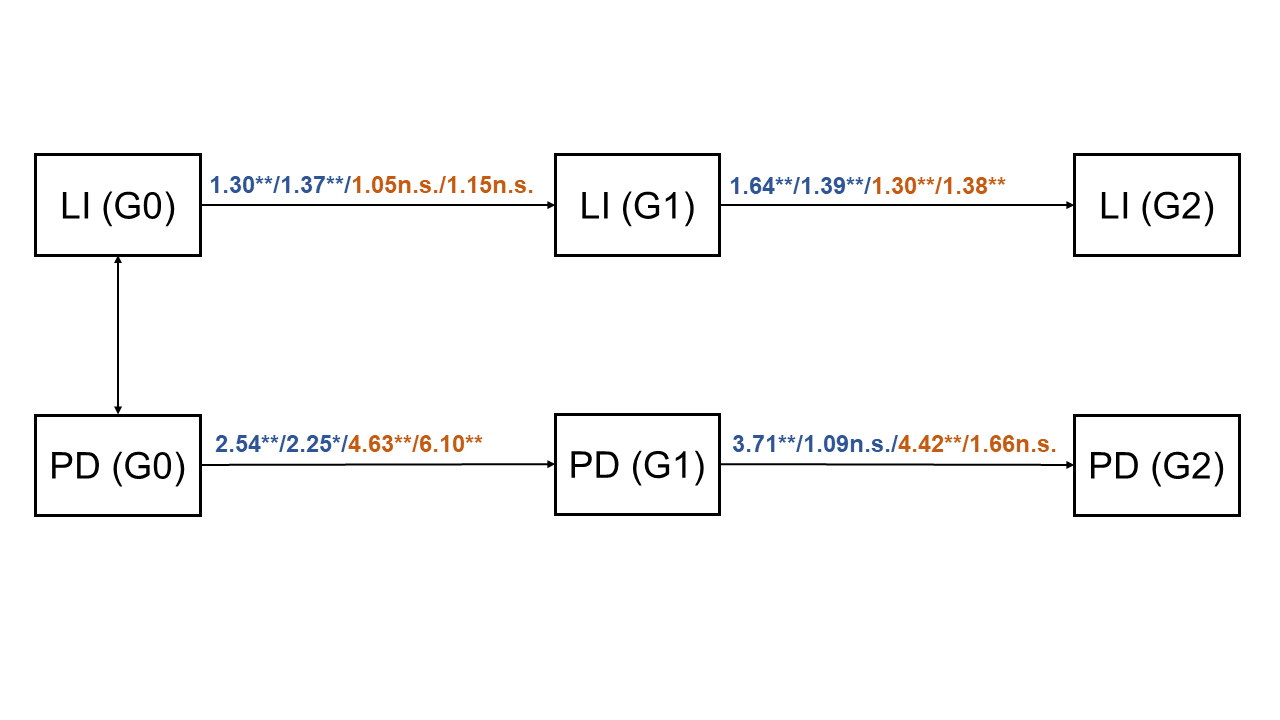


Model 2


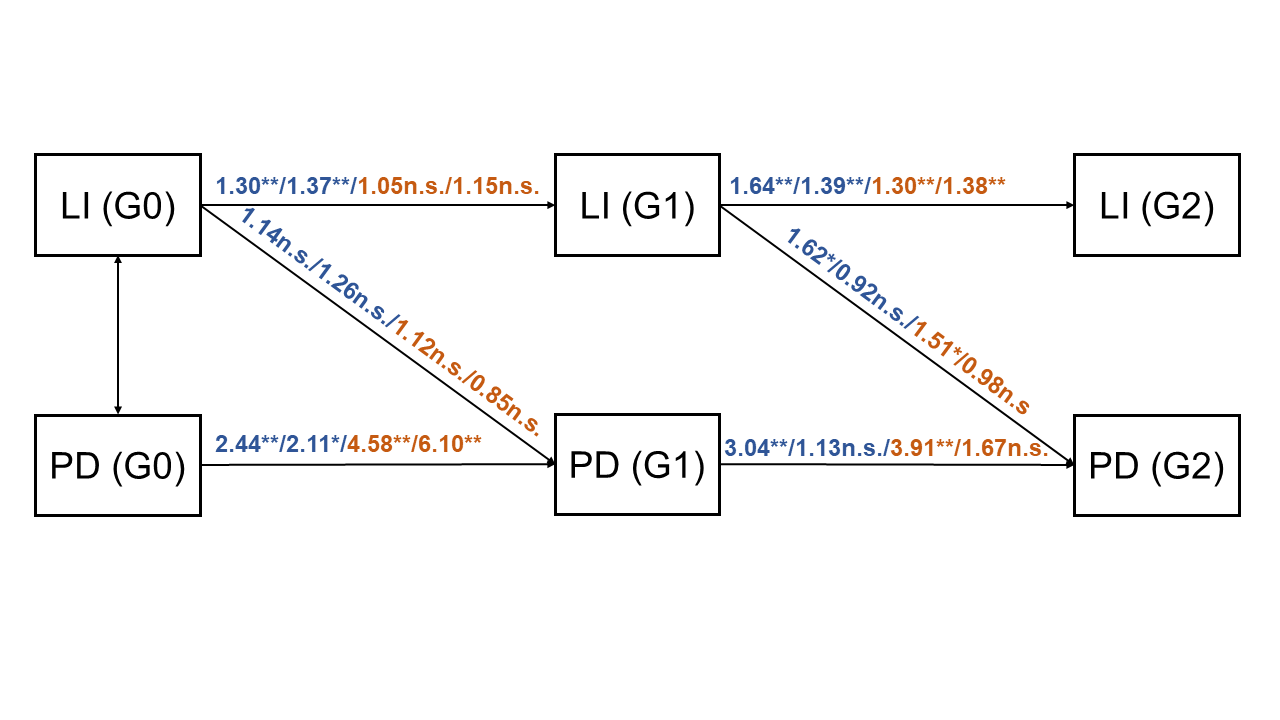


Model 3


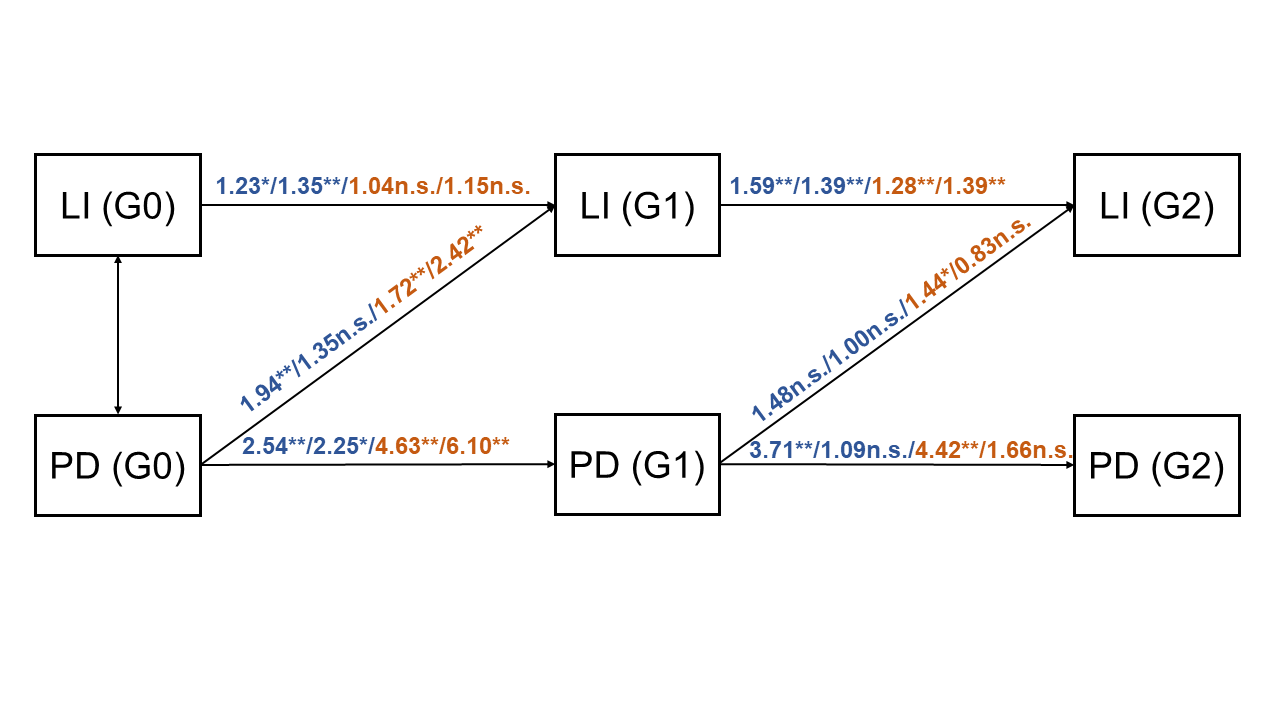


Model 4


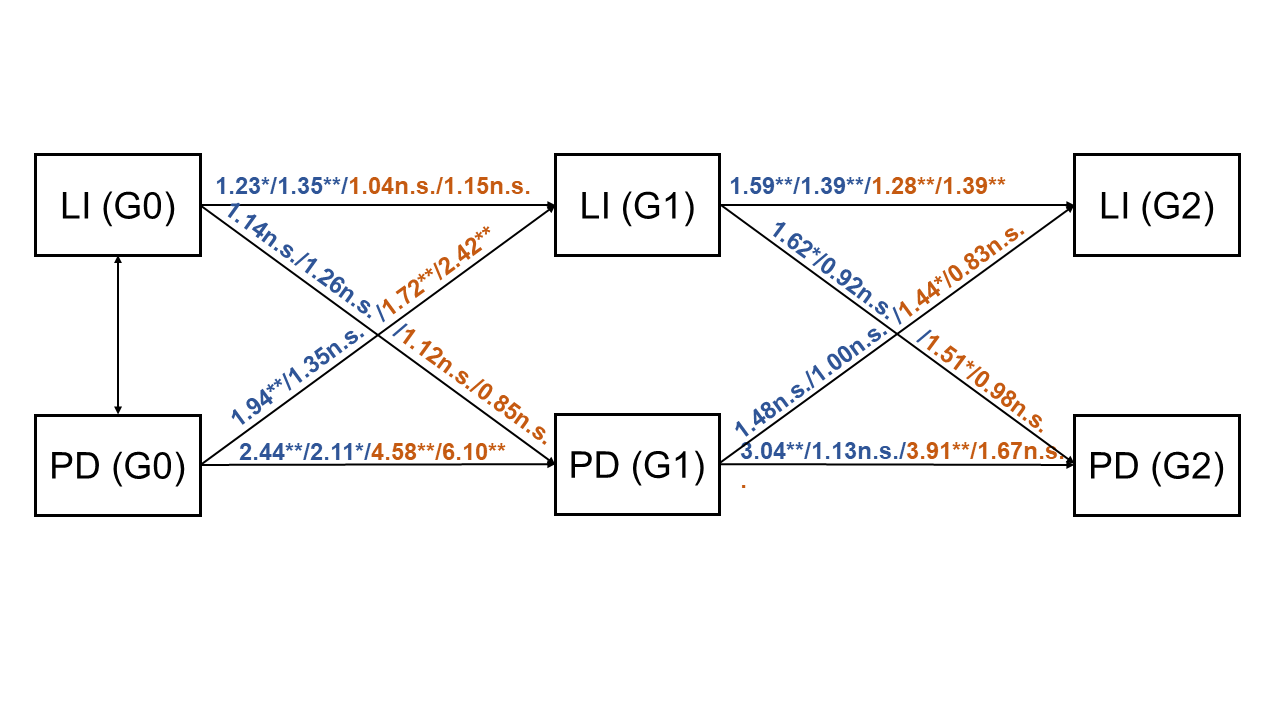


Note: Covariates are omitted from the figure. Presented estimates are exponentiated coefficients (odds ratios) for patriline-G2 men/patriline-G2 women/matriline-G2 men/matriline-G2 women in each path. LI: Low Income, PD: Psychiatric Disorders. n.s.: not significant; * p<0.05; ** p<0.01. Model 1 includes autoregressive paths reflecting transmission of low income and psychiatric disorders, respectively, from G0 to G1 and from G1 to G2. Model 2 additionally includes cross-lagged paths going from low income to psychiatric disorders across generations. Model 3 additionally includes cross-lagged paths going from psychiatric disorders to low income across generations. Model 4 encompasses all paths specified in the previous models.

**Figure A2. Sensitivity analysis, structural equation models for the multigenerational transmissions of low income and psychiatric disorders, by lineage and grandchild gender, for sample without income imputation for G2.**


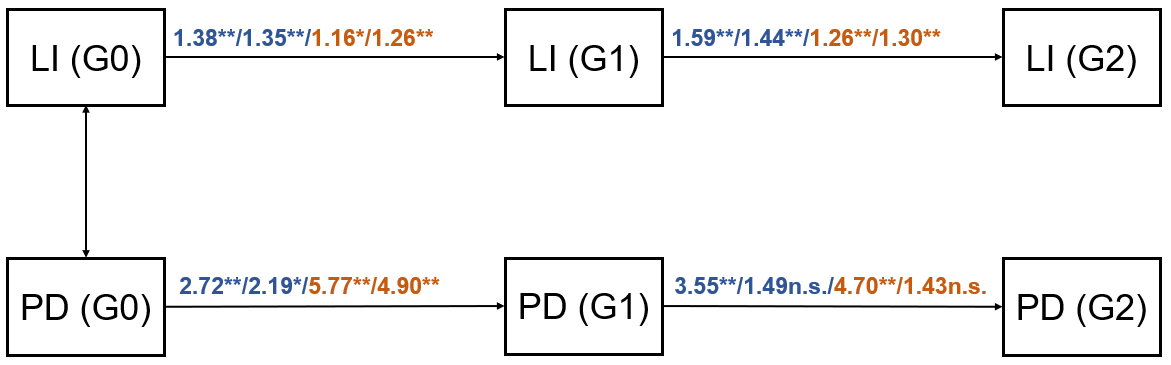


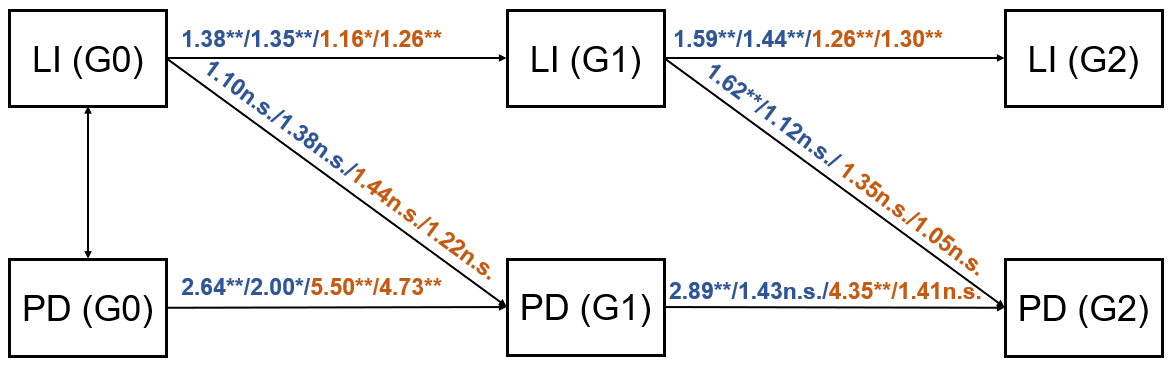


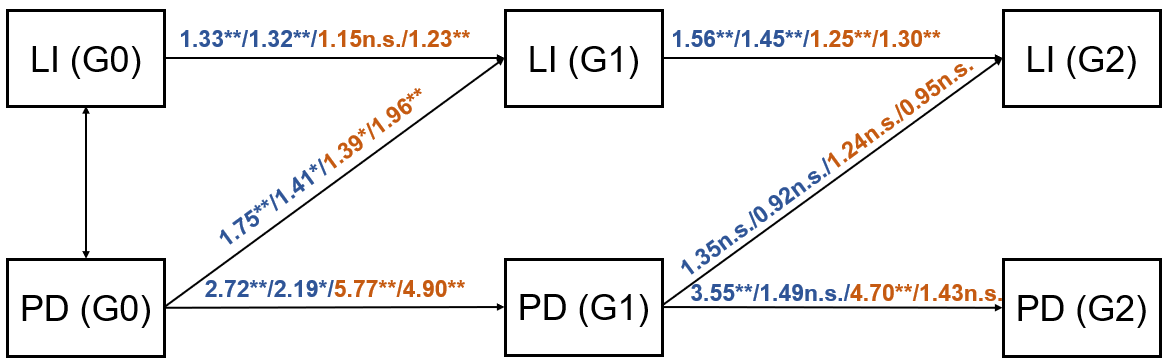


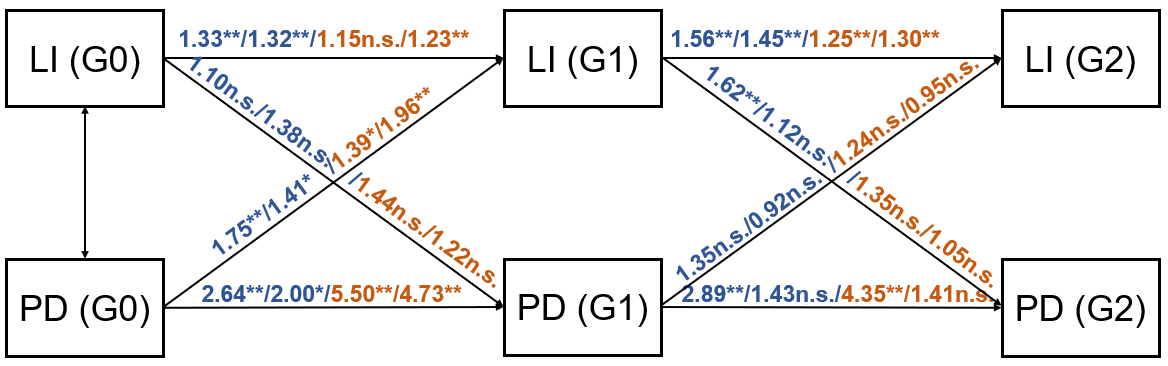


Note: Covariates are omitted from the figure. Presented estimates are exponentiated coefficients (odds ratios) for patriline-G2 men/patriline-G2 women/matriline-G2 men/matriline-G2 women in each path. LI: Low Income, PD: Psychiatric Disorders. n.s.: not significant; * p<0.05; ** p<0.01. Model 1 includes autoregressive paths reflecting transmission of low income and psychiatric disorders, respectively, from G0 to G1 and from G1 to G2. Model 2 additionally includes cross-lagged paths going from low income to psychiatric disorders across generations. Model 3 additionally includes cross-lagged paths going from psychiatric disorders to low income across generations. Model 4 encompasses all paths specified in the previous models.

**Figure A3. Sensitivity analysis, structural equation models for the multigenerational transmissions of low income and psychiatric disorders, by lineage and grandchild gender, for sample with alternative coding strategy for G0 women.**
